# The Uremic Toxin Adsorbent AST-120 Abrogates Cardiorenal Injury Following Myocardial Infarction

**DOI:** 10.1371/journal.pone.0083687

**Published:** 2013-12-13

**Authors:** Suree Lekawanvijit, Sirinart Kumfu, Bing H. Wang, Minako Manabe, Fuyuhiko Nishijima, Darren J. Kelly, Henry Krum, Andrew R. Kompa

**Affiliations:** 1 Centre of Cardiovascular Research and Education in Therapeutics, Department of Epidemiology and Preventive Medicine, Monash University, Melbourne, Australia; 2 Department of Medicine, University of Melbourne, St. Vincent’s Hospital, Melbourne, Australia; 3 Department of Pathology, Faculty of Medicine, Chiang Mai University, Chiang Mai, Thailand; 4 Cardiac Electrophysiology Research and Training Center, Faculty of Medicine, Chiang Mai University, Chiang Mai, Thailand; 5 Pharmaceutical Department, Kureha Corporation, Tokyo, Japan; University of Central Florida, United States of America

## Abstract

An accelerated progressive decline in renal function is a frequent accompaniment of myocardial infarction (MI). Indoxyl sulfate (IS), a uremic toxin that accumulates from the early stages of chronic kidney disease (CKD), is contributory to both renal and cardiac fibrosis. IS levels can be reduced by administration of the oral adsorbent AST-120, which has been shown to ameliorate pathological renal and cardiac fibrosis in moderate to severe CKD. However, the cardiorenal effect of AST-120 on less severe renal dysfunction in the post-MI setting has not previously been well studied. MI-induced Sprague-Dawley rats were randomized to receive either AST-120 (MI+AST-120) or were untreated (MI+Vehicle) for 16 weeks. Serum IS levels were measured at baseline, 8 and 16 weeks. Echocardiography and glomerular filtration rate (GFR) were assessed prior to sacrifice. Renal and cardiac tissues were assessed for pathological changes using histological and immunohistochemical methods, Western blot analysis and real-time PCR. Compared with sham, MI+Vehicle animals had a significant reduction in left ventricular ejection fraction (by 42%, p<0.001) and fractional shortening (by 52%, p<0.001) as well as lower GFR (p<0.05) and increased serum IS levels (p<0.05). A significant increase in interstitial fibrosis in the renal cortex was demonstrated in MI+Vehicle animals (p<0.001). Compared with MI+Vehicle, MI+AST-120 animals had increased GFR (by 13.35%, p<0.05) and reduced serum IS (p<0.001), renal interstitial fibrosis (p<0.05), and renal KIM-1, collagen-IV and TIMP-1 expression (p<0.05). Cardiac function did not change with AST-120 treatment, however gene expression of TGF-β1 and TNF-α as well as collagen-I and TIMP-1 protein expression was decreased in the non-infarcted myocardium (p<0.05). In conclusion, reduction of IS attenuates cardio-renal fibrotic processes in the post-MI kidney. KIM-1 appears to be a sensitive renal injury biomarker in this setting and is correlated with serum IS levels.

## Introduction

Coexistence of renal and cardiac dysfunction, known as cardiorenal syndrome, has an adverse impact on clinical outcomes following acute myocardial infarction (MI). Approximately one third of hospitalized MI patients present with coexisting kidney dysfunction [1] and one fifth develop worsening renal function during hospitalization [2]. These patients are at higher risk for in-hospital death [3,4] and cardiovascular events (hospitalization for congestive heart failure, recurrent MI and stroke) after discharge as well as short- and long-term mortality [2-5]. Even in post-MI patients with mild renal impairment, which may be transient, 10-year prognosis is still poor [2]. 

Despite clear clinical evidence, the pathophysiology that underlies the development and progression of renal impairment following MI is not well understood. We recently demonstrated in an experimental model of MI that worsening renal function occurs early post-MI, may be transient and is strongly related to activation of renal inflammatory-fibrosis pathways which lead to nonreversible functional impairment [6]. Expression of kidney injury molecule (KIM)-1, a novel biomarker of kidney injury, appears to be a promising biomarker to detect and monitor post-MI renal injury [6]. 

Indoxyl sulfate (IS), a protein-bound uremic toxin which accumulates when renal excretory function is impaired, has been demonstrated to be cardio-[7] and reno-toxic [8,9] by enhancing organ fibrosis. This toxin is of clinical importance especially in severe kidney disease, as its removal by current conventional hemodialysis is severely limited. However, accumulation of IS is also observed in the early stages of chronic kidney disease [10]. Given its harmful biological effects, early intervention may be required to limit progression to end-stage renal disease. AST-120, an oral adsorbent, is an IS-reducing agent which has been reported to prevent IS-induced renal [11] and cardiac [12] interstitial fibrosis in the setting of moderate to severe chronic kidney disease. Whether AST-120 has beneficial effects in cardiorenal syndrome in post-MI patients with early stage CKD is currently unknown. 

We measured circulating (plasma) levels of IS at different time points post-MI in stored plasma samples from a previous rat MI study [6]. A significant increase in IS levels was observed at 12 and 16 weeks in MI compared with sham animals, whilst renal functional impairment was observed at 16 but not 12 weeks [6]. In this study we therefore investigated the effect of AST-120 on reducing IS-associated cardiorenal toxicity focusing on cardiorenal fibrosis in a 16-week post-MI model with secondary renal dysfunction. 

## Materials and Methods

### Study design

Male Sprague-Dawley rats (220-250 g) underwent left anterior descending (LAD) ligation to induce myocardial infarction (MI) on day 1 (D1) [13]. Briefly, animals were intubated and artificially ventilated with 2% isofluorane in oxygen. A left thoractomy was performed and the LAD coronary artery ligated with a 6–0 prolene suture a few millimeters below its origin. Visible blanching and hypokinesis of the anterior LV wall and swelling of the left atrium were indicative of successful ligation. A sham operation involved the same procedure except the LAD was not ligated. The thorax was then closed after briefly inflating the lungs, and the skin sutured. .

MI animals were then randomized after a full recovery to receive either AST-120 (MI + AST-120, n= 14) or no treatment (MI + vehicle, n= 17) for 16 weeks. AST-120 (Kremezin^®^, Kureha Pharmaceuticals, Tokyo, Japan) was administrated post-operatively in the chow at 8% w/w. Sham operated rats (n= 13) were used as controls.

**Figure 1:**
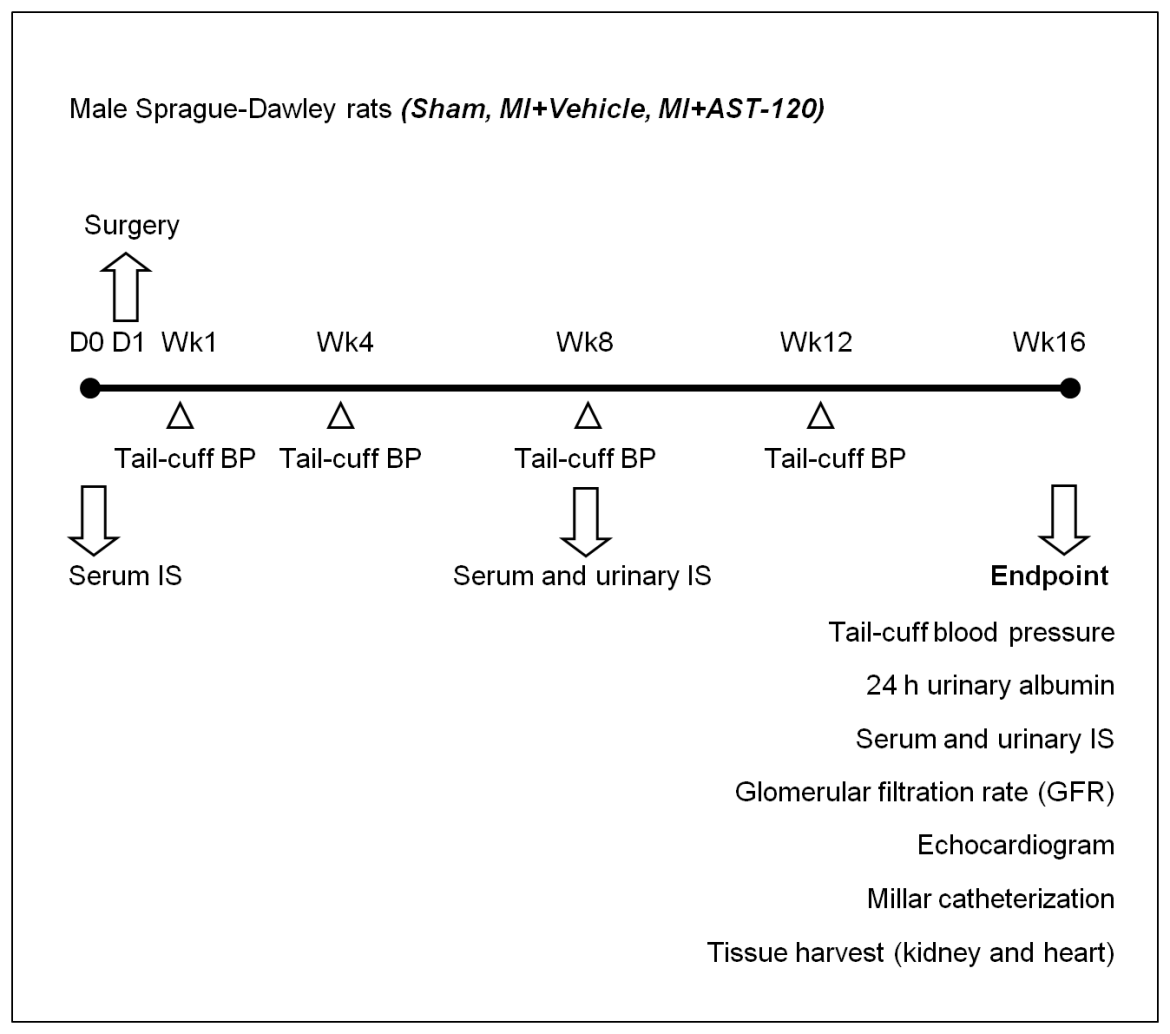
Experimental design.

The experimental design is shown in Figure 1. Serum IS levels were measured using high performance liquid chromatography method (Shimadzu, Kyoto, Japan) at baseline (1 day before surgery, Day 0 – D0), 8 weeks and endpoint (16 weeks post-op); and urine IS at 8 and 16 weeks. Systolic blood pressure (BP) was measured in conscious rats using the tail-cuff method at 1, 4, 8, 12 and 16 weeks post-MI. In the final week, glomerular filtration rate (GFR) was assessed, animals placed in metabolic cages for urine collection and analysis, echocardiography performed for assessment of cardiac function, and Millar catheterization was performed to obtain hemodynamics and pressure-volume loops prior to tissue harvest. Tissues were assessed for pathological and molecular changes using histological methods, Western blot analysis and real-time PCR.

The investigation conformed with the Guide for the Care and Use of Laboratory Animals published by the US National Institutes of Health (PHS Approved Animal Welfare Assurance no. A5587-01). All animal usage was also approved by St Vincent’s Hospital’s Animal Ethics Committee (AEC) in accordance with National Health and Medical Research Council (NHMRC) guide for the care and use of laboratory animals. 

### Cardiac function Assessment – Echocardiography and Millar catheterization

Echocardiography was performed in lightly anaesthetized animals (ketamine 40 mg/kg, xylazine 5 mg/kg, i.p.) using a Vivid 7 (GE Vingmed, Horten, Norway) echocardiography machine with a 10MHz phased array probe. The procedure was performed as per published protocol routinely used in our laboratory [14].

For Millar pressure-volume loop assessment animals were anesthetized with pentobarbitone (60 mg/kg, i.p.) and intubated for cardiac catheterization procedures, as previously described [15]. Briefly, under positive pressure ventilation a 2F miniaturized combined catheter/micromanometer (Model SPR838 Millar instruments, Houston, TX) was inserted into the right common carotid artery to obtain aortic blood pressure and then advanced into the left ventricle to obtain left ventricular pressure-volume (PV) loops. PV loops were recorded at steady state and during transient preload reduction, achieved by occlusion of the inferior vena cava and portal vein with the ventilator turned off and animal apnoeic. The PV loops were assessed using Millar conductance data acquisition and analysis software PVAN 3.2. 

### Renal function assessment

#### Glomerular filtration rate (GFR)

GFR was performed prior to sacrifice to measure kidney function. Briefly, animals were intravenously injected with a radioactive isotope, ^99^technetium-diethylene triamine penta-acetic acid (^99^Tc-DTPA), excreted solely by the glomerulus [16]. The DTPA was prepared at a rate of 37 MBq/mL (1mCi/mL), and 0.26mL injected into each rat. Animals were bled 43 minutes after injection and their plasma radioactivity was measured to evaluate the rate of DTPA excretion, this was compared with the counts of the standard reference prepared at the time of injection [17]. The calculated GFR was corrected for body weight recorded before the procedure and reported as GFR/kg. 

#### 24-hour urine albumin

Urine samples obtained from metabolic caging were stored at -20°C for measurement of urine albumin. A double antibody radioimmunoassay was used, as previously described [18].

### Histological study

Hearts and kidneys were removed after Millar catheterization, weighed, fixed in 10% neutral buffered formalin and then processed for histopathology and immunohistochemistry. 

#### Infarct size assessment

Cross sections of left ventricle (LV) of all MI animals were stained with Picrosirius Red and scanned (Aperio, Aperio Technologies Inc., Vista, CA) for infarct size. Infarct size was reported in animals with transmural infarction as the averaged percentage of the endocardial and epicardial scarred circumference of the LV [19]. 

#### Quantitation of matrix deposition

Ten random non-overlapping fields from the renal cortex-to-corticomedullary region (glomeruli excluded) of all animals were captured using a microscope attached to a digital camera (Carl Zeiss AxioVision, Germany). The proportional area of Picrosirius red-stained matrix was calculated using image analysis (AIS, Analytical imaging Station Version 6.0, Imaging Research Inc, Ontario, Canada). Focal interstitial fibrosis/scarring, defined as an increase in matrix deposition in interstitial spaces that is distinguishable from the surrounding area, was reported as descriptive data and not included in the quantitative analysis. Since morphological abnormalities were virtually absent in the renal medulla and image analysis of matrix deposition in this area was severely interfered by retained red blood cells in medullary vasa recta, the renal medulla was excluded from analysis in the present study. 

Matrix deposition in non-infarct LV myocardium was evaluated using the same method described above. 

#### Kidney injury molecule-1 (KIM-1) expression

Tissue expression of kidney KIM-1 was assessed by immunohistochemistry [20], using goat antiKIM-1 (R&D systems, Minneapolis, MN, 1:200 dilution) antibody. Numbers of KIM-1 positive tubules were counted from whole kidney sections. 

### Western blot analysis

Renal cortical tissue and non-infarcted myocardial tissue (30 mg) was homogenised with 1 ml of modified RIPA buffer in the presence of protease and phosphatase inhibitors. Equal amounts of protein (30 µg) were separated by 10% sodium dodecyl sulfate–polyacrylamide gel electrophoresis, and electrophoretically transferred to nitrocellulose membranes (Amersham Biosciences). Western blot analysis was performed as per manufacturer’s protocol with specific antibodies (TGF-β, phospho-Smad2, Smad2, phospho-p44/42, p44/42, phospho-p38, p38, phospho-SAPK/JNK, SAPK/JNK, phospho-NF-κB p65, and NF-κB p65 antibodies – Cell Signaling Technology, Beverly, MA, USA; collagen-III – Biogenex, Fremont, CA, USA; collagen- I, collagen-IV, tissue inhibitor of metalloproteinase I (TIMP-1) – Novus Biologicals, Littleton, CO, USA; pan-actin antibody – NeoMarkers, Fremont, CA, USA) and then visualized by enhanced chemiluminescence (Thermo Scientific, Rockford, IL, USA). Band intensity was analysed using ImageJ software (NCBI). Pan-actin and total-protein antibodies were used as endogenous controls for non-phosphorylated proteins and corresponding phosphorylated-proteins, respectively. 

### Quantitative mRNA Expression

Total RNA was extracted from 30 mg renal cortical tissue using Qiagen RNeasy kits (Qiagen, Hilden, Germany). Messenger RNA was reverse transcribed and triplicate cDNA aliquots were amplified using sequence-specific primers (Geneworks, Adelaide, SA, Australia) with SYBR Green detection (Applied Biosystems) using an ABI prism 7900HT sequence Detection System (Applied Biosystems). Real-time polymerase chain reaction (PCR) was used to quantify pro-fibrotic [transforming growth factor-beta 1 (TGF-β1), connective tissue growth factor (CTGF)] and pro-inflammatory cytokine [tumor necrosis factor (TNF-α), interleukin-1 beta (IL-1β) and IL-6] gene expression. The primer pairs were designed using Primer Express 2.0 software (Applied Biosystems) based on published sequences (http://www.ncbi.nlm.nih.gov). 18S rRNA was used as an endogenous control in all experiments to correct for the expression of each gene.

Quantitative mRNA expression of non-infarct myocardial tissue was performed using the same method as for renal tissue to evaluate pro-fibrotic (TGF-β1, CTGF, collagen-I, collagen-III), pro-hypertrophic [atrial natriuretic peptide (ANP), beta-myosin heavy chain (β-MHC), alpha-skeletal muscle actin (α-SkM-Ac)], and pro-inflammatory cytokine (TNF-α, IL-6, IL-1β) gene expression. 18S rRNA was used as an endogenous control in all experiments to correct for the expression of each gene.

### Statistical analysis

Data are presented as mean ± SEM. One-way ANOVA with Bonferroni's multiple comparison test or Kruskal-Wallis test with Dunn's multiple comparison test were used for comparisons among all groups for parametric and non-parametric data, respectively. For comparisons between 2 groups, unpaired Student t-test was used for parametric data and Mann Whitney test for non-parametric data. All statistical analyses were performed using GraphPad Prism 5. A two-tailed p-value of less than 0.05 was considered statistically significant. 

## Results

The total number of animals used in this study was 44 (13 sham, 17 MI+Vehicle and 14 MI+AST-120). Overall post-operative mortality, exclusive within 2 days after LAD ligation, was 31.6%. 

There was no difference in infarct size between MI+Vehicle and MI+AST-120 groups (41.6% vs 43.8%) (Table 1). 

**Table 1:** Infarct size, tissue weights and indoxyl sulfate levels.

	**Sham** (n = 13)	**MI + Vehicle** (n = 17)	**MI + AST-120** (n = 14)
**Infarct size** (%)	–	41.6	43.8
**Body weight** (g)	546.7 ± 12.42	538.1 ± 12.35	521.4 ± 16.31
**Organ weight**			
HW/BW (g/kg)	2.47 ± 0.06	2.74 ± 0.07**	2.83 ± 0.10*
LV/BW (g/kg)	1.77 ± 0.05	1.94 ± 0.06*	2.03 ± 0.06*
Lung/BW (g/kg)	2.87 ± 0.08	3.03 ± 0.06	3.10 ± 0.08
Kidney/BW (g/kg)	6.45 ± 0.18	6.76 ± 0.16	6.59 ± 0.15
**Serum indoxyl sulfate** (mg/dL)			
Week 0 (Baseline)	0.10 ± 0.02	0.10 ± 0.01	0.10 ± 0.2
Week 8	0.19 ± 0.02	0.21 ± 0.01	0.04 ± 0.004***,***^†††^***
Week 16 (Endpoint)	0.19 ± 0.02	0.26 ± 0.03*	0.03 ± 0.004***,***^†††^***
**Change in serum indoxyl sulfate**			
(**Endpoint - Baseline**) (mg/dL)	0.08 ± 0.01	0.17 ± 0.03*	-0.07 ± 0.02***,***^†††^***
**24-h urinary indoxyl sulfate** (mg)			
Week 8	3.6 ± 0.2	4.2 ± 0.2	0.8 ± 0.1***,***^†††^***
Week 16	4.4 ± 0.3	4.2 ± 0.3	0.8 ± 0.1***,***^†††^***

Data are presented as mean ± SEM.

*p<0.05, **p<0.01, ***p<0.001 vs Sham. ^†††^p<0.001 vs MI+Vehicle.

HW/BW - heart weight/body weight; LV/BW - left ventricular weight/body weight.

The heart weight/body weight (HW/BW) and left ventricular weight/body weight (LV/BW) were significantly greater in both MI groups compared with sham animals (Table 1). There was no difference in BW, lung/BW and kidney/BW across the groups at 16 weeks post-MI (Table 1).

### Serum and urinary indoxyl sulfate levels

There was no difference in serum IS levels among groups at baseline (Table 1). Compared with sham, serum IS levels in the MI+Vehicle group increased by 10.27% at week 8 (ns) and became significant at week 16 by 29.52% (Table 1). Change in serum IS levels (deltaIS (△IS) = Endpoint – Baseline serum IS) was significantly greater in MI+Vehicle animals (Table 1). Treatment with AST-120 decreased absolute serum IS levels at weeks 8 and 16 post-MI as well as △IS; p<0.001). 

Twenty-four hour urinary IS excretion was significantly reduced by AST-120 at weeks 8 and 16 compared with MI+Vehicle animals (Table 1). 

### Cardiac function and hemodynamic assessment

#### Echocardiographic study

Compared with sham, ejection fraction and fractional shortening was reduced by 42% and 52%, respectively (p<0.001), and isovolumetric relaxation time (IVRT) was increased by 25% (p<0.05) in MI+Vehicle animals (Table 2). There was a non-significant improvement in IVRT, a marker of diastolic function, with AST-120 treatment (50% reduction, p=0.12). Treatment with AST-120 had no significant effects on systolic dysfunction. 

**Table 2:** Blood pressure, cardiac function and renal function assessment.

	**Sham** (n = 13)	**MI + Vehicle** (n = 17)	**MI + AST-120** (n = 14)
**Systolic blood pressure** (mmHg)			
Week 1	132 ± 3	122 ± 2*	121 ± 2*
Week 4	137 ± 1	129 ± 3	130 ± 4
Week 8	142 ± 4	147 ± 3	140 ± 3
Week 12	144 ± 5	143 ± 5	135 ± 3
Week 16	149 ± 7	146 ± 5	153 ± 3
**Echocardiographic study**			
LVEF (%)	66 ± 2	38 ± 3***	42 ± 3***
FS (%)	40 ± 2	19 ± 2***	22 ± 2***
LV mass (g/m^2^)	1.7 ± 0.1	1.8 ± 0.1	1.7 ± 0.1
IVRT (msec)	24 ± 2	30 ± 2*	27 ± 2
Deceleration time (msec)	35 ± 2	36 ± 2	36 ± 1
E/A ratio	2.2 ± 0.3	2.5 ± 0.4	2.8 ± 0.5
E/E' ratio	0.24 ± 0.01	0.26 ± 0.01	0.27 ± 0.02
**Pressure-volume assessment**			
CAP (mmHg)	74 ± 3	72 ± 3	74 ± 3
HR (beat/min)	276 ± 13	284 ± 11	277 ± 14
LVESP (mmHg)	95 ± 3	89 ± 2	91 ± 3
dP/dt_max_ (mmHg/s)	5480 ± 135	4366 ± 153***	4663 ± 128***
PRSW (mmHg)	88 ± 7	59 ± 5**	63 ± 5**
τ Logistic (msec)	9.6 ± 0.2	13 ± 0.9**	13 ± 0.7**
τ Weiss-in steady state (msec)	12.8 ± 0.3	17.0 ± 0.9***	18 ± 1.4*
LVEDP (mmHg)	4.2 ± 0.2	6.2 ± 0.4***	6.9 ± 0.7***
-dP/dt_min_ (mmHg/s)	4981 ± 176	3223 ± 148***	3513 ± 146***
**Glomerular filtration rate** (ml/min/kg)	10.56 ± 0.34	9.15 ± 0.53*	9.81 ± 0.33
**24-hour albuminuria** (mg)	0.41 ± 0.08	0.88 ± 0.24	0.38 ± 0.08 (p=0.08 vs MI+Vehicle)

Data are presented as mean ± SEM.

*p<0.05, **p<0.01, ***p<0.001 vs Sham.

LVEF – left ventricular ejection fraction; FS - fractional shortening; LV mass - left ventricular mass; IVRT - isovolumic relaxation time; CAP - central aortic pressure; HR - heart rate; LVESP - LV end systolic pressure; dP/dt_max_ - rate of LV pressure rise; PRSW - preload recruitable stroke work; τ (Tau) - load independent measure of isovolumetric relaxation time; LVEDP - LV end diastolic pressure; dP/dt_min_ - rate of LV pressure fall.

#### Systolic tail-cuff BP

At 1 week post-MI, both MI groups had significantly lower tail-cuff BP compared with sham animals (Table 2). BP was comparable between MI groups at all time points measured.

#### Left ventricular pressure-volume assessment

A significant reduction in the slope of the preload recruitable stroke work (PRSW) relationship (p<0.01) and dP/dt_max_ (p<0.001) was observed in both MI groups compared with sham animals (Table 2). 

MI animals had a significant prolongation of τ Logistic (p<0.01) and τ Weiss (p<0.05). Compared with sham, higher left ventricular end diastolic pressure (LVEDP) and lower dP/dt_min_ were demonstrated in both MI groups (p<0.001, Table 2). 

There was no difference in all measured parameters between MI+Vehicle and MI+AST-120 groups. 

### Renal function assessment

#### Endpoint GFR

GFR was significantly decreased in MI+Vehicle compared with sham animals at 16 weeks post-MI (10.56 vs 9.15 ml/min/kg) (Table 2). A 47% improvement, calculated by 100*[(difference in GFR between MI+AST-120 and MI+Vehicle groups which is 0.66)/( difference in GFR between sham and MI+Vehicle groups which is 1.41)], in GFR was observed in MI+AST-120 group (p=0.31).

#### 24-hour urine albumin

A 2.12-fold increase in albuminuria was observed in MI+Vehicle compared with sham animals (p>0.05) (Table 2). AST-120 normalized albuminuria (back to sham levels) but this was not statistically significant (p=0.08). 

### Cardiac tissue studies

#### Cardiac interstitial matrix deposition

Cardiac interstitial fibrosis in non-infarct myocardium was significantly increased by 59% in MI+Vehicle compared with sham animals (p<0.01; Table 3). AST-120 did not reduce interstitial cardiac fibrosis. 

**Table 3:** Fibrosis and gene expression data.

	**Sham** (n = 13)	**MI + Vehicle** (n = 17)	**MI + AST-120** (n = 14)
**Cardiac interstitial fibrosis** (% area)	1.44 ± 0.15	2.29 ± 0.22**	2.34 ± 0.15***
**Cardiac gene expression** (expressed as a ratio relative to 18s)			
Transforming growth factor-β1	4.42 ± 0.19	5.56 ± 0.40**	4.39 ± 0.35^†^
Connective tissue growth factor	0.77 ± 0.09	2.18 ± 0.40***	2.16 ± 0.43**
Collagen-I	1.49 ± 0.14	2.95 ± 0.69*	2.18 ± 0.41
Collagen-III	1.85 ± 0.20	3.16 ± 0.57*	2.43 ± 0.53
Tumor necrosis factor-α	4.05 ± 0.30	6.07 ± 0.95*	3.59 ± 0.54^†^
Interleukin-6	0.30 ± 0.15	0.95 ± 0.46	0.61 ± 0.25
Atrial natriuretic peptide	0.09 ± 0.04	0.33 ± 0.07**	0.31 ± 0.07*
β-myosin heavy chain	0.42 ± 0.04	0.79 ± 0.07***	0.66 ± 0.08*
α -skeletal muscle actin	0.18 ± 0.02	0.40 ± 0.06***	0.33 ± 0.05*
**Renal interstitial fibrosis** (% area)	1.87 ± 0.21	4.14 ± 0.13***	3.47 ± 0.21***,***^†^***
**Renal gene expression** (expressed as a ratio relative to 18s)			
Transforming growth factor-β1	7.72 ± 0.95	8.37 ± 0.98	9.00 ± 0.80
Connective tissue growth factor	15.38 ± 2.09	14.57 ± 2.33	17.64 ± 1.97
Tumor necrosis factor-α	7.95 ± 1.03	8.97 ± 1.53	9.53 ± 1.22

Data are presented as mean ± SEM.

*p<0.05, **p<0.01, ***p<0.001 vs Sham. ^†^p<0.05 vs MI+Vehicle.

#### Cardiac gene expression

**Fibrotic markers:** TGF-β1, CTGF, collagen-I and collagen-III mRNA expression in non-infarct myocardium was significantly increased in MI+Vehicle compared with sham animals (Table 3). AST-120 treatment reduced TGF-β1 mRNA expression back to sham levels (p<0.05). There was a non-significant decrease in collagen-I (by 52.74%) and collagen-III (by 55.78%) mRNA expression in MI+AST-120 animals (Table 3). 

**Inflammatory markers:** MI+Vehicle animals showed a significant increase in expression of cardiac TNF-α gene that was normalized by AST-120 (p<0.05) (Table 3). Cardiac IL-6 gene expression showed no significant difference among the groups (Table 3). Expression of cardiac IL-1β mRNA expression was too low to be detected.

**Hypertrophic markers:** MI showed a significant increase in expression of ANP, β-MHC and α-SkM-Ac mRNA compared with sham animals however no effect was observed with AST-120 treatment (Table 3).

#### Cardiac protein expression

**Figure 2:**
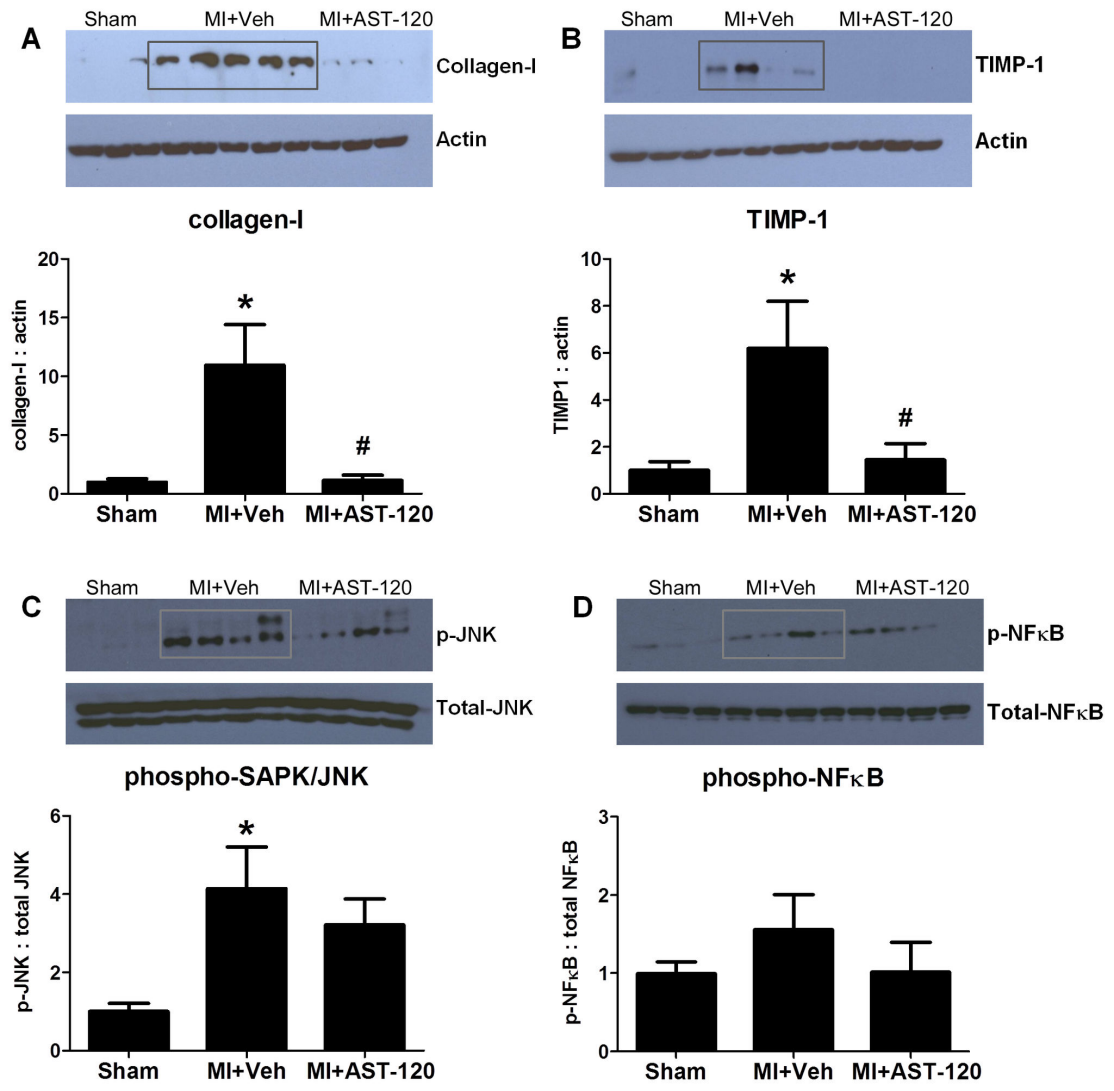
Cardiac protein expression. MI+Vehicle animals showed an increase in the myocardial expression of collagen-1, TIMP-1 and phospho-SAPK/JNK (A, B, C). A significant reduction in collagen-I and TIMP-1 expression was observed with AST-120 treatment (A, B). An increase in phospho-NFκB expression (D, p>0.05) is also normalized by AST-120 (p>0.05). *p<0.05 vs Sham. **^*#*^**p<0.05 vs MI+Vehicle.

A significant increase in cardiac protein expression of collagen-I and TIMP-1 (p<0.05) in MI+Vehicle animals was significantly normalized by AST-120 treatment (p<0.05, Figure 2A,B). 

 An increase in phospho-SAPK/JNK (p<0.05, Figure 2C) but not phospho-NFκB was observed in MI+Vehicle animals. AST-120 did not affect the levels of these proteins (Figure 2C,D).

### Renal tissue studies

#### Renal interstitial matrix deposition (fibrosis)

Focal tubulointerstitial scarring with/without inflammatory cell infiltration was observed in 4 out of 17 MI+Vehicle animals whilst absent in MI+AST-120 and sham animals. The lesions were mainly located in the cortex and corticomedullary junction similar to that observed in our previous study [6].

An increase in diffuse interstitial fibrosis was observed in the cortex of both MI groups compared with sham animals (p<0.001) (Table 3). Treatment with AST-120 significantly reduced renal interstitial fibrosis (by 30%, calculated by 100*[(difference between MI+AST-120 and MI+Vehicle groups which is 0.67)/( difference between sham and MI+Vehicle groups which is 2.27)], p<0.05). 

#### Renal gene expression

Gene expression of fibrotic markers TGF-β1 and CTGF remained unchanged between the 3 groups at 16 weeks post-MI (Table 3). Expression of the inflammatory cytokine TNF-α was also unchanged between the groups (Table 3), and other cytokines such as IL-6 and IL-1β were not detected in the tissue samples.

#### Renal protein expression

**Figure 3:**
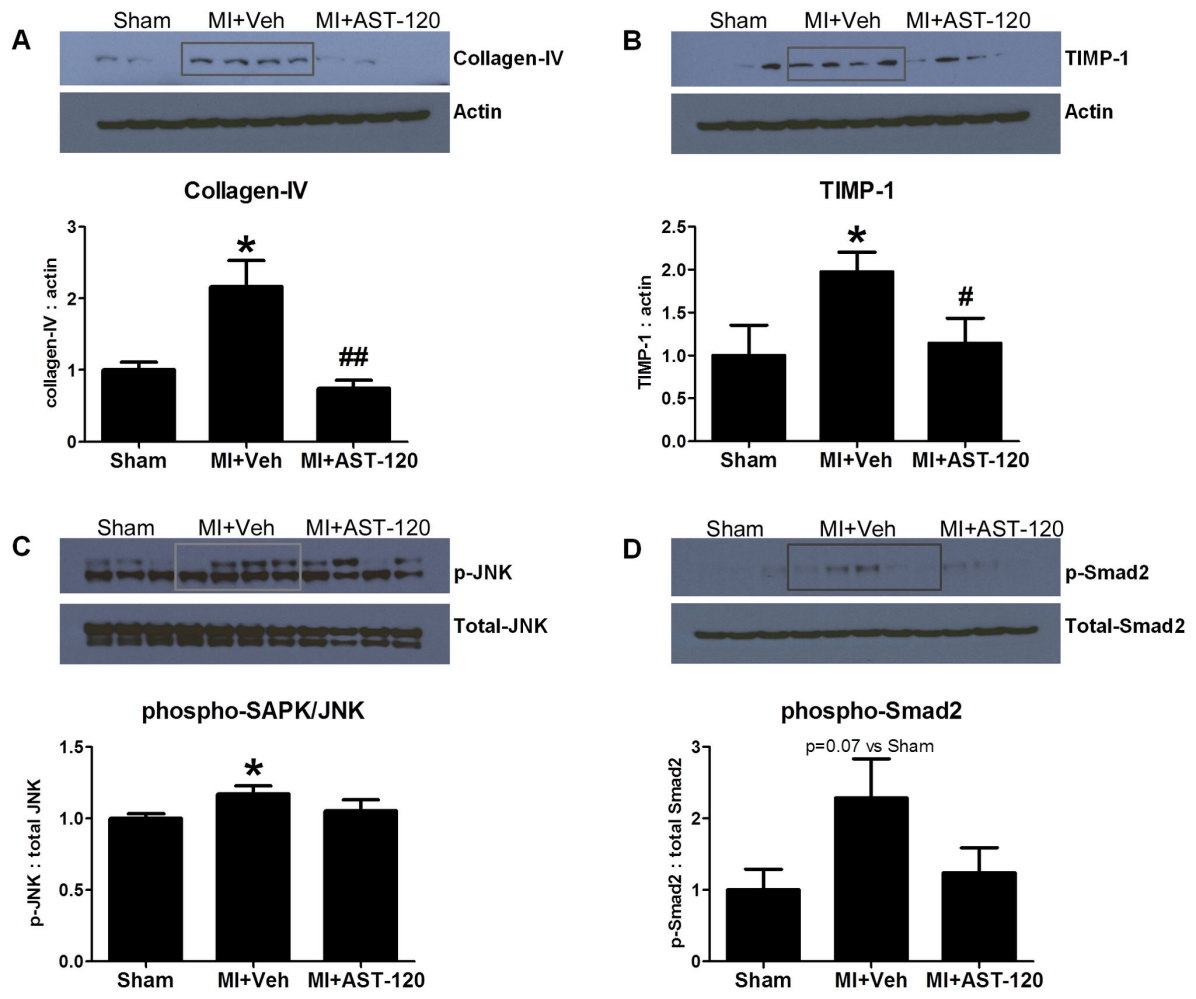
Renal protein expression. MI+Vehicle animals significantly increased renal protein expression of collagen-IV, TIMP-1 and phospho-SAPK/JNK (A, B, C) and a trend toward an increase in renal phospho-Smad2 expression (D). AST-120 treatment significantly reduced collagen-IV and TIMP-1 expression (A, B). A trend toward decreased phospho-SAPK/JNK and phospho-Smad2 expression was also observed (C, D). *p<0.05 vs Sham. **^*#*^**p<0.05, ^##^p<0.01 vs MI+Vehicle.

Renal protein expression of collagen-IV and TIMP-1was significantly increased in MI+Vehicle animals (p<0.05). AST-120 significantly normalized collagen-IV and TIMP-1 expression (p<0.05, Figure 3A,B). Phospho-SAPK/JNK was significantly increased (p<0.05) and a non-significant increase in phospho-Smad2 protein level (p=0.07) was also observed in MI+Vehicle animals. AST-120 treatment indicated a trend toward sham levels of expression of these proteins (Figure 3C,D).

#### Kidney injury molecule-1 (KIM-1) expression

**Figure 4:**
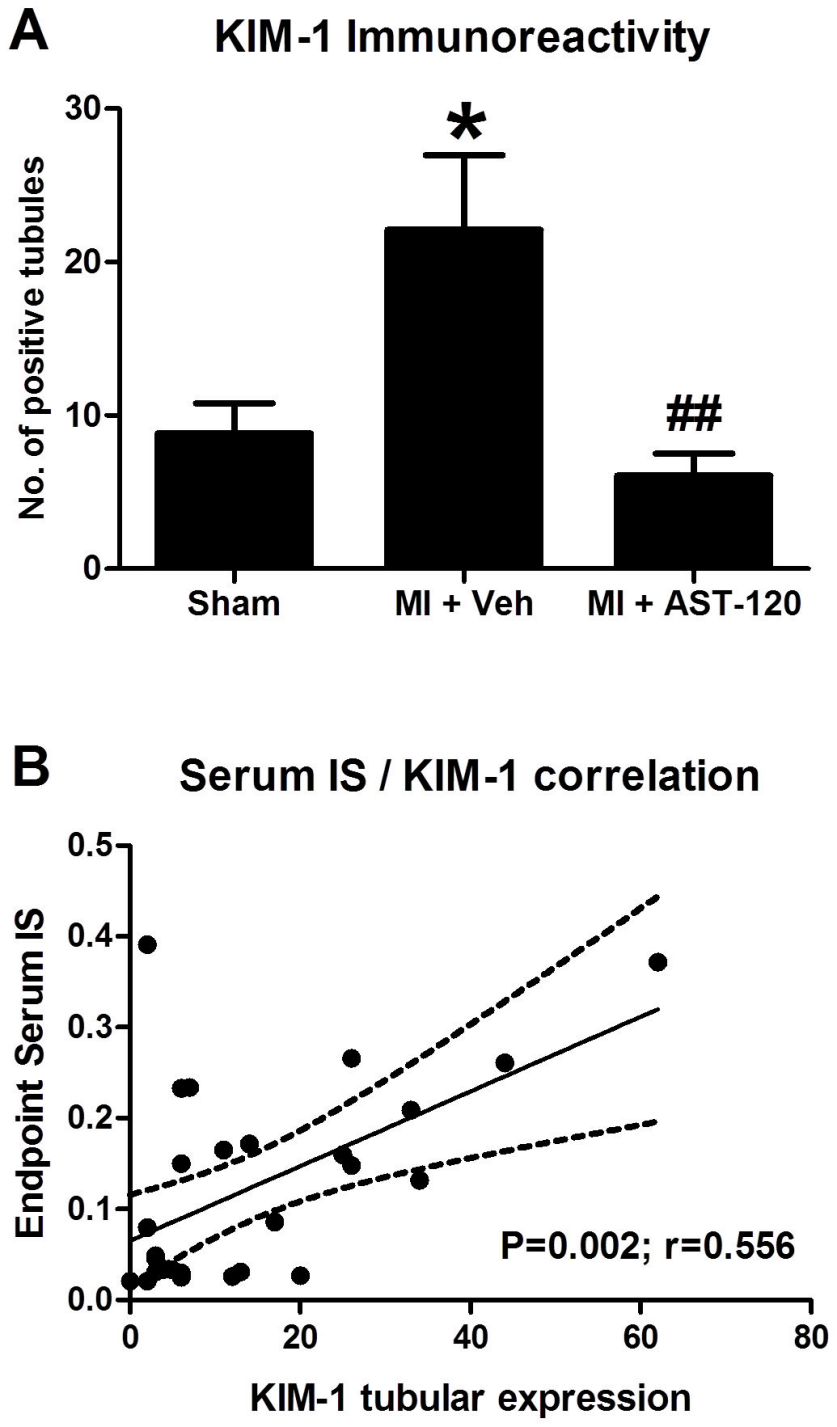
Renal tubular expression of kidney injury molecule-1 (KIM-1) and correlation with serum IS levels. Increased tubular expression of KIM-1 was observed in MI+Vehicle animals, AST-120 treatment significantly reduced KIM-1 expression to sham levels (A). Serum IS was significantly and positively correlated with KIM-1 expression (B, p<0.01), *p<0.05 vs Sham. ^##^p<0.01 vs MI+Vehicle.

Compared with sham, MI+Vehicle animals showed an increase in the number of KIM-1 positively stained tubules (p<0.05). AST-120 reduced renal KIM-1 expression (p<0.05) compared with MI+Vehicle animals (Figure 4A). Furthermore, KIM-1 expression was positively correlated with serum IS levels (r=0.56; p=0.002; Figure 4B).

## Discussion

The present study has demonstrated that MI animals develop cardiac and renal dysfunction in association with an elevation in serum IS levels at 16 weeks post-MI. This was accompanied by increased cardiorenal interstitial fibrosis. Increased collagen and TIMP-1 protein expression in the myocardium and kidney, increased cardiac TGF-β1 and TNF-α gene expression and increased renal KIM-1 protein expression were significantly attenuated by AST-120 treatment (together with reduced serum IS levels), despite no significant improvement of renal or cardiac function. 

Hemodynamic derangements post-MI may in part be contributory to long-term renal dysfunction as reported in our previous study [6]. Transient decline in systolic blood pressure following MI is associated with early worsening of renal function and may activate the renal fibrogenesis pathway leading to permanent renal dysfunction [6]. 

A significant elevation of serum IS levels was observed in MI+Vehicle animals together with a decrease in GFR by 13.35%. Given that a GFR over 90 ml/min/1.73m^2^ is considered normal in man unless there is evidence of kidney disease [21], a 13.35% decrease is equivalent to a GFR decline to 78 from 90 ml/min/1.73m^2^. With histological evidence of kidney damage observed, renal impairment following MI in the present study is equivalent to moving from stage 1 to stage 2 CKD according to the National Kidney Foundation practice guidelines for chronic kidney disease [21]. A similar finding showing IS accumulation in early stages of CKD has been reported in patients with diabetic nephropathy who have estimated GFR 60-89 ml/min/1.73m^2^ or stage 2 CKD (*p*=0.077) [10]. 

Beneficial renal and cardiovascular effects of lowering IS levels by AST-120 have been demonstrated in both CKD patients and animal models [12,22-24]. However, this has been mainly observed in primary kidney disease with at least moderate to severe renal dysfunction. Studies using AST-120 in less severe CKD are very rare, particularly in the setting of MI or heart failure in which renal complications are secondary rather than the primary phenomena. To our knowledge, there is only one small clinical study (n=20) demonstrating beneficial effects of AST-120 treatment in heart failure patients with moderate CKD [24]. In that uncontrolled study, patients who received AST-120 (in combination with standard medications) had an improvement in renal function, atrial natriuretic peptide levels, heart failure signs/symptoms (i.e. edema and cardiothoracic ratio), length of hospital stay and number of admissions after a 2-year follow-up period compared with prior to treatment [24].

In the present study, AST-120 appears to be more beneficial to the kidney than heart as the improvements were observed at both the structural and protein expression levels in the kidney, whilst improvements were only observed at the protein and gene expression levels in the heart. This may be because the kidney is the initial organ exposed to accumulating levels of IS, thereby being more susceptible to direct injury caused by the toxin. On the other hand, treatment by reducing IS is likely to show its effects on improving renal fibrosis before cardiac fibrosis. Of note, the follow-up analysis after 16-week treatment may be beyond the peak of renal pro-inflammatory and pro-fibrotic gene expression in response to the MI event. 

Reactive fibrosis in non-infarcted myocardium occurs due to ongoing cardiac remodeling following MI. This may in part be due to increased IS secondary to post-MI renal dysfunction enhancing the remodeling process, as AST-120 normalized collagen I protein expression, and fibrotic (TGF-β1) and inflammatory (TNF-α) gene expression in the present study. These factors are well-described as being involved in the progression of cardiac remodeling [25-27]. A reduction in cardiac gene expression of collagen I and III was also observed in non-infarcted myocardium despite not reaching statistical significance. 

In the kidney, AST-120 was demonstrated to significantly prevent renal fibrosis. Although we did not observe functional improvement, a trend toward improved GFR and albuminuria was observed. Follow-up time may not have been sufficient to achieve the maximum effects of AST-120. Similarly in the heart, beneficial effects of AST-120 on structural remodeling in addition to its effect in suppressing cardiac expression of proteins and genes involved in the inflammation-fibrosis process may need a longer follow-up period. 

Increased TIMP-1, a key factor involved in extracellular matrix degradation, protein expression in both heart and kidney observed in the present study is likely related to IS levels since its expression was significantly normalized by reducing IS levels with AST-120 treatment. Renal TIMP-1 mRNA expression previously demonstrated an additional increase following administration of IS in an experimental CKD model associated with accelerated progression of renal interstitial fibrosis compared with CKD animals without IS administration [9]. Reducing IS accumulation in CKD models is also associated with a reduction of renal TIMP-1 expression and interstitial fibrosis [11]. 

Postulated IS-mediated mechanisms of cardiorenal fibrosis relate to the reactive oxygen species/NF-κB/TGF-β1 pathway [12,28,29]. Although a reduction in increased NF-κB protein expression with AST-120 did not reach statistical significance, expression levels in the myocardium were completely normalized by AST-120 treatment (Figure 2D). This may be explained by the relatively mild severity of post-MI renal dysfunction requiring a longer time for IS-mediated cardiorenal injury to peak in the MI+Vehicle animals. A similar explanation can be put forward for phospho-SAPK/JNK which has been demonstrated to be downstream of TGF-β1-mediated renal fibrosis [30]. 

Early detection of post-MI renal injury is highly crucial because even mild and transiently worsening renal function confers poor clinical outcomes as previously outlined [2]. Serum creatinine, a surrogate for glomerular filtration may reflect renal damage once substantial parenchyma has been lost [31]. In contrast, KIM-1, a transmembrane protein highly expressed on the surface of injured renal tubular epithelial cells [32], has been demonstrated to be correlated with tubulointerstitial fibrosis in patients with various kidney diseases [33] as well as in a rat MI model with renal dysfunction [6]. The present study has also demonstrated a positive correlation between KIM-1 expression in renal tubules and serum IS levels. This suggests that KIM-1 may also represent a marker of IS-induced kidney injury post-MI.

### Study limitations

The present study did not include a RAAS blocker, either as comparator or in combination with AST-120, since we wished to first conduct a proof-of-concept study examining the direct contribution of uremic toxins to cardiorenal fibrosis as well as the potential therapeutic implications of reducing serum IS levels in this model of type 2 cardiorenal syndrome (defined by chronic cardiac dysfunction causing progressive CKD).

## Conclusions

Post-MI renal impairment is equivalent to moving from stage 1 to stage 2 CKD and associated with accumulation of the uremic toxin IS. Reducing IS levels with AST-120 inhibits activation of cardiorenal fibrosis pathways despite no functional improvement occuring with either organ over the time period studied. Thus, IS appears to be implicated, at least in part, in the progression of some of the structural features of type 2 cardiorenal syndrome and AST-120 might potentially represent useful adjunctive therapeutic strategy in this clinical setting following MI. 
